# Cardiac cephalalgia: a case series of four patients and updated literature review

**DOI:** 10.1186/s12245-022-00436-2

**Published:** 2022-07-29

**Authors:** Hitoshi Kobata

**Affiliations:** Osaka Mishima Emergency Critical Care Center, 11-1 Minamiakutagawa-cho Takatsuki, Osaka, 569-1124 Japan

**Keywords:** Cardiac cephalalgia, Cardiac cephalgia, Acute myocardial ischemia, Thunderclap headache, Neurological Emergency

## Abstract

**Background:**

Cardiac damage is common in patients with acute brain injury; however, little is known regarding cardiac-induced neurological symptoms. In the International Classification of Headache, Third Edition (ICHD-III), cardiac cephalalgia is classified as a headache caused by impaired homeostasis.

**Methods:**

This report presents four patients with acute myocardial infarction (AMI) who presented with headache that fulfilled the ICHD-III diagnostic criteria for cardiac cephalalgia. A systematic review of cardiac cephalalgia using the Preferred Reporting Items for Systematic Reviews and Meta-analyses guidelines is also presented.

**Results:**

Case 1: A 69-year-old man with a history of percutaneous coronary intervention (PCI) developed sudden severe occipital pain, nausea, and cold sweating. Coronary angiography (CAG) revealed occlusion of the right coronary artery (RCA). Case 2: A 66-year-old woman complained of increasing occipitalgia and chest discomfort while riding a bicycle. CAG demonstrated 99% stenosis of the left anterior descending artery. Case 3: A 54-year-old man presented with faintness, cold sweating, and occipitalgia after eating lunch. CAG detected occlusion of the RCA. Case 4: A 72-year-old man went into shock after complaining of a sudden severe headache and nausea. Vasopressors were initiated and emergency CAG was performed, which detected three-vessel disease. In all four, electrocardiography (ECG) showed ST segment elevation or depression and echocardiography revealed a left ventricular wall motion abnormality. All patients underwent PCI, which resulted in headache resolution after successful coronary reperfusion. A total of 59 cases of cardiac cephalalgia were reviewed, including the four reported here. Although the typical manifestation of cardiac cephalalgia is migraine-like pain on exertion, it may present with thunderclap headache without a trigger or chest symptoms, mimicking subarachnoid hemorrhage. ECG may not always show an abnormality. Headaches resolve after successful coronary reperfusion.

**Conclusions:**

Cardiac cephalalgia resulting from AMI can present with or without chest discomfort and even mimic the classic thunderclap headache associated with SAH. It should be recognized as a neurological emergency and treated without delay.

**Supplementary Information:**

The online version contains supplementary material available at 10.1186/s12245-022-00436-2.

## Background

The interaction between the brain and the heart is an emerging area of clinical interest. Cardiac damage is common in patients with acute brain injury. Neurogenic stress cardiomyopathy (also known as neurogenic stunned myocardium) is widely recognized in patients with acute neurological disease [1]; however, little is known regarding cardiac-induced neurological symptoms. In 1997, Lipton et al. reported two cases of exertional headache associated with myocardial ischemia; based on these and a review of five similar previous ones, they coined the term “cardiac cephalgia” (“cardiac cephalalgia” in the current classification) [2]. In the International Classification of Headache, Third Edition (ICHD-III) [3], cardiac cephalalgia is classified as a headache caused by impaired homeostasis (Table 1). Cardiac cephalalgia is described as migraine-like headache that occurs during an episode of myocardial ischemia and is usually aggravated by exercise. The diagnosis can be challenging because cardiac cephalalgia is uncommon and the headache is not always associated with exertion; headache may occur at rest without chest symptoms [4–6]. Only a few reported cases of cardiac cephalalgia presented with sudden severe headache (thunderclap headache), which mimics subarachnoid hemorrhage (SAH) [7–10]. Both myocardial ischemia and SAH are potentially life-threatening; therefore, early recognition with appropriate treatment is critically important. Consequently, it is essential to understand the characteristics of cardiac cephalalgia as a neurological emergency and accurately diagnose it to enable appropriate intervention. This report presents four patients diagnosed with cardiac cephalalgia and reviews the relevant literature to summarize the disease characteristics and current evidence regarding the diagnosis and treatment of this uncommon clinical entity.

**Table 1:** Diagnostic criteria of cardiac cephalalgia

A. Any headache fulfilling criterion C
B. Acute myocardial ischemia has been demonstrated
C. Evidence of causation demonstrated by at least two of the following:
1. headache has developed in temporal relation to the onset of acute myocardial ischemia
2. either or both of the following:
a) headache has significantly worsened in parallel with worsening of the myocardial ischemia
b) headache has significantly improved or resolved in parallel with improvement in or resolution of the myocardial ischemia
3. headache has at least two of the following four characteristics:
a) moderate to severe intensity
b) accompanied by nausea
c) not accompanied by phototophia or phonophobia
d) aggravated by exertion
4. headache is relieved by nitroglycerine or derivatives of it
D. Not better accounted for by another ICHD-3 diagnosis

## Methods

### Cases

**Fig. 1:**
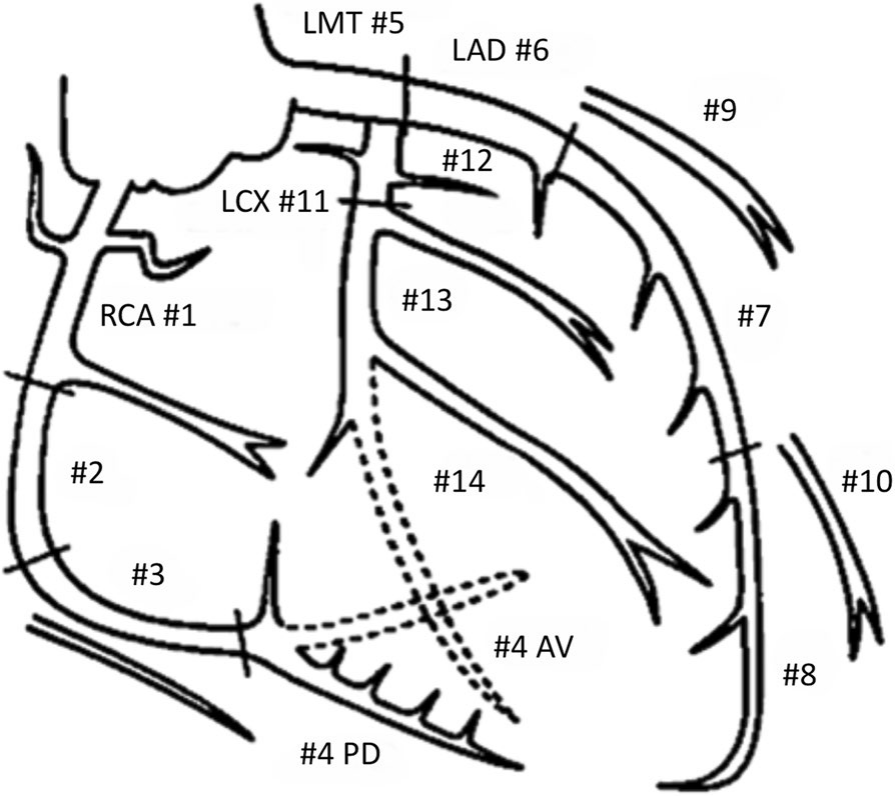
Coronary artery segments according to the American Heart Association classification [11]. RCA, right coronary artery; LCA, left coronary artery; LMT, left main trunk; LAD, left anterior descending coronary artery; LCx, left circumflex coronary artery; AV, atrioventricular nodal artery; PD, posterior descending coronary artery

Since 2009, Osaka Mishima Emergency Critical Care Center has experienced four cases of headache that fulfilled the ICHD-III diagnostic criteria for cardiac cephalalgia. Characteristics of the four patients are summarized in Table 2 and briefly described below. Coronary artery lesions are described using the American Heart Association classification (Fig. 1) [11].

**Table 2:** Patient characteristics

Case	Age	Sex	Site	Quality	Intensity	Onset	Autonomic signs	Cardiac symptoms	Trigger	ECG	Echocardiogram findings	Coronary lesion	Therapy	Follow-up
1	69	M	Occipital- right shoulder	Pulsatile	Severe	Sudden	Nausea, cold sweating	None	None	ST elev in II, III, aVF	Inf wall akinesis	RCA (#2) 100%	PCI (RCA)	Resolved
2	66	F	Occipital	NA	Severe	Sudden	Cold sweating	Chest discomfort	Bicycle	ST elev in II, III, aVF, V1-4	Takotsubo	LAD (#7) 99%	Heparin, PCI (LAD)	Resolved
3	54	M	Posterior neck-occipital	Strangulation	Moderate	Gradual	Cold sweating, faintness	Chest discomfort	Meal	ST elev in II, II, aVF, III AV block	Inf wall akinesis	RCA (#3) 100%, LAD (#7) 90%, LCX (#13) 100%	PCI (RCA)	Resolved
4	72	M	Headache	NA	Severe	Sudden	Nausea, vomiting	None	None	ST elev in aVR, II, III, aVF, ST dep in V2-5	Lat, post, inf wall akinesis, ant-septal severe hypokinesis, mitral regurgitation	RCA (#3) 99%, LCX (#11) 99%, LAD (75%)	PCI (RCA), IABP, ECMO	Resolved/Died

*ECG* electrocardiography, *M* male, *F* female, *NA* not available, *elev* elevation, *inf* inferior, *lat* lateral, *post* posterior, *ant* anterior, *RCA* right coronary artery, *LAD* left anterior descending artery, *LCX* left circumflex artery, *PCI* percutaneous coronary intervention, *IABP* intra-aortic balloon pumping, *ECMO* extracorporeal membranous oxygenation

### Literature review

The PubMed (National Center for Biotechnology Information, National Institutes of Health, Bethesda, MD, USA) and Scopus (Elsevier, Amsterdam, Netherlands) databases were searched in accordance with the Preferred Reporting Items for Systematic Reviews and Meta-analyses guidelines [12]. The terms "cardiac cephalalgia" OR "cardiac cephalgia" OR "headache and acute coronary syndrome" OR "headache and myocardial infarction" OR "anginal headache" were used without a publication year limitation. The references of each publication were also reviewed to find other potentially relevant reports. Only full-text English language studies were included. Duplicated patients were excluded.

## Results

### Case presentations

#### Case 1

A 69-year-old man with a 50-year history of smoking presented with sudden onset severe pulsatile occipitalgia during sleep. The headache was described as the worst in his life and was accompanied by nausea, cold sweating, and a five-minute episode of unconsciousness. He had a history of hypothyroidism and percutaneous coronary intervention (PCI) in the left anterior descending artery (LAD) and the right coronary artery (RCA) for angina. Electrocardiography (ECG) in the ambulance during transport to the hospital showed ST elevation in leads II and III.

**Fig. 2:**
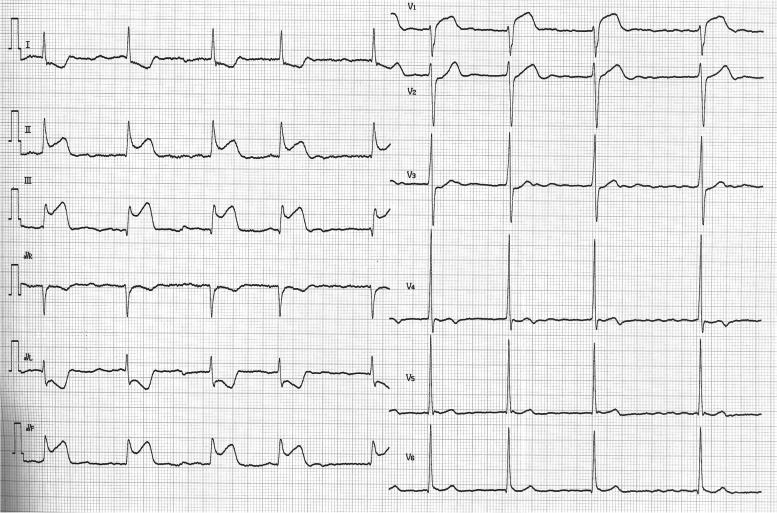
Electrocardiogram of case 1 shows ST segment elevation in leads II, III, and aVF

**Fig. 3:**
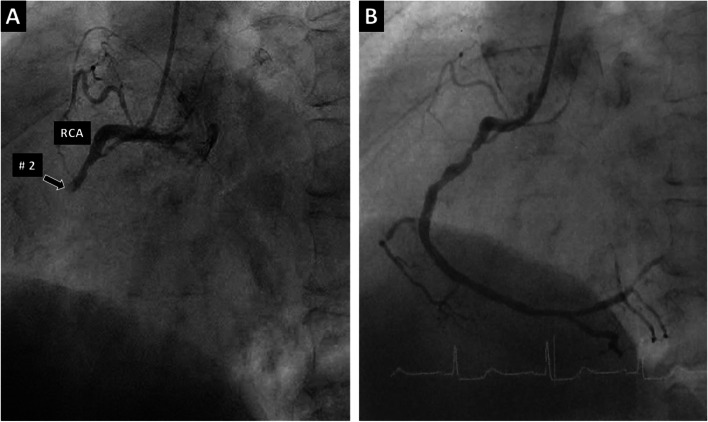
Coronary angiogram of case 1 before **A** and after **B** percutaneous coronary intervention with stenting to segment 2. The occluded right coronary artery was recanalized

He was alert on arrival complaining of severe occipitalgia but no chest pain. Blood pressure was 132/70 mm Hg and heart rate was 57 beats per minute (bpm). Emergency head computed tomography (CT) showed no abnormalities. ECG showed ST segment elevation in leads II, III, and aVF (Fig. 2). Echocardiography revealed akinesis of the inferior wall of the left ventricle. Blood chemistry studies showed no elevation of creatine kinase–myocardial band (CKMB) concentration (0.6 ng/mL; reference range, < 3.6 ng/mL). When repeatedly asked if he had any chest symptoms, he admitted to having slight chest discomfort. Emergency coronary angiography (CAG) was subsequently performed and acute myocardial infarction (AMI) was diagnosed. The angiogram revealed occlusion of segment 2 in the RCA (Fig. 3) and 75% stenosis of segment 12 in the left circumflex artery (LCX). During PCI for the occluded RCA, he went into ventricular fibrillation (VF), which recovered to sinus rhythm after electrical defibrillation. His headache subsided after treatment and he was discharged uneventfully 11 days later.

#### Case 2

A 66-year-old woman with a history of rheumatoid arthritis presented with sudden occipitalgia while riding a bicycle. The pain was moderate and gradually intensified over time. While resting, she reported chest discomfort and cold sweating. ECG during emergency transportation to the hospital showed ST segment elevation in leads V3–V5. On arrival, she was alert and profusely sweating. Blood pressure was 90/40 mm Hg and heart rate was 60 bpm. ECG showed ST segment elevation in leads II, III, aVF, and V2–V4. CKMB was normal (1.4 ng/mL) and troponin T was negative. Emergency head CT showed no intracranial hemorrhage. CT angiography disclosed a basilar–left superior cerebellar artery aneurysm 2 mm in diameter; no bleb was visualized. Magnetic resonance imaging of the brain confirmed no hemorrhage and no arterial dissection. Therefore, the aneurysm was considered unruptured. Echocardiography revealed takotsubo-like abnormal movement with an ejection fraction of 30%.

She was initially treated with intravenous heparin. Although her headache subsided soon after admission, CKMB concentration the next day was 501.1 ng/mL. She underwent CAG 15 days later after cardiac function had been restored. A 99% stenosis was found in LAD segment 7 and stents were placed. She was discharged home uneventfully. Magnetic resonance (MR) angiography of the brain nine months later showed no change in aneurysmal size or shape.

#### Case 3

A 54-year-old man with a 30-year history of smoking presented with faintness, cold sweating, and nausea after eating lunch, followed by strangulating occipitalgia. He was taking medications for hyperlipidemia, hypertension, and diabetes. ST segment elevation was seen in leads II and III on ECG during transportation to the hospital. Upon arrival, he complained of moderate occipitalgia but no chest pain. Blood pressure was 66/38 mm Hg and heart rate was 44 bpm. ECG showed ST segment elevation in leads II, III, and aVF. Echocardiography showed hypokinesis of the inferior wall. His headache subsided while being evaluated in the emergency room. CKMB concentration was elevated (22.8 ng/mL) and troponin T was positive; therefore, AMI was diagnosed. After initiation of vasopressors and temporary pacing, emergency CAG was performed, which showed occlusion of the RCA segment 3 and LCX segment 13, as well as 90% stenosis of the LAD segment 7. PCI was performed for the RCA, which was thought to be the culprit lesion. After hemodynamic stabilization, he underwent PCI for the LAD stenosis 15 days later. The LCX was considered a chronic occlusion and was not treated. He had no further headaches after the initial PCI and he was discharged 21 days later uneventfully.

#### Case 4

**Fig. 4:**
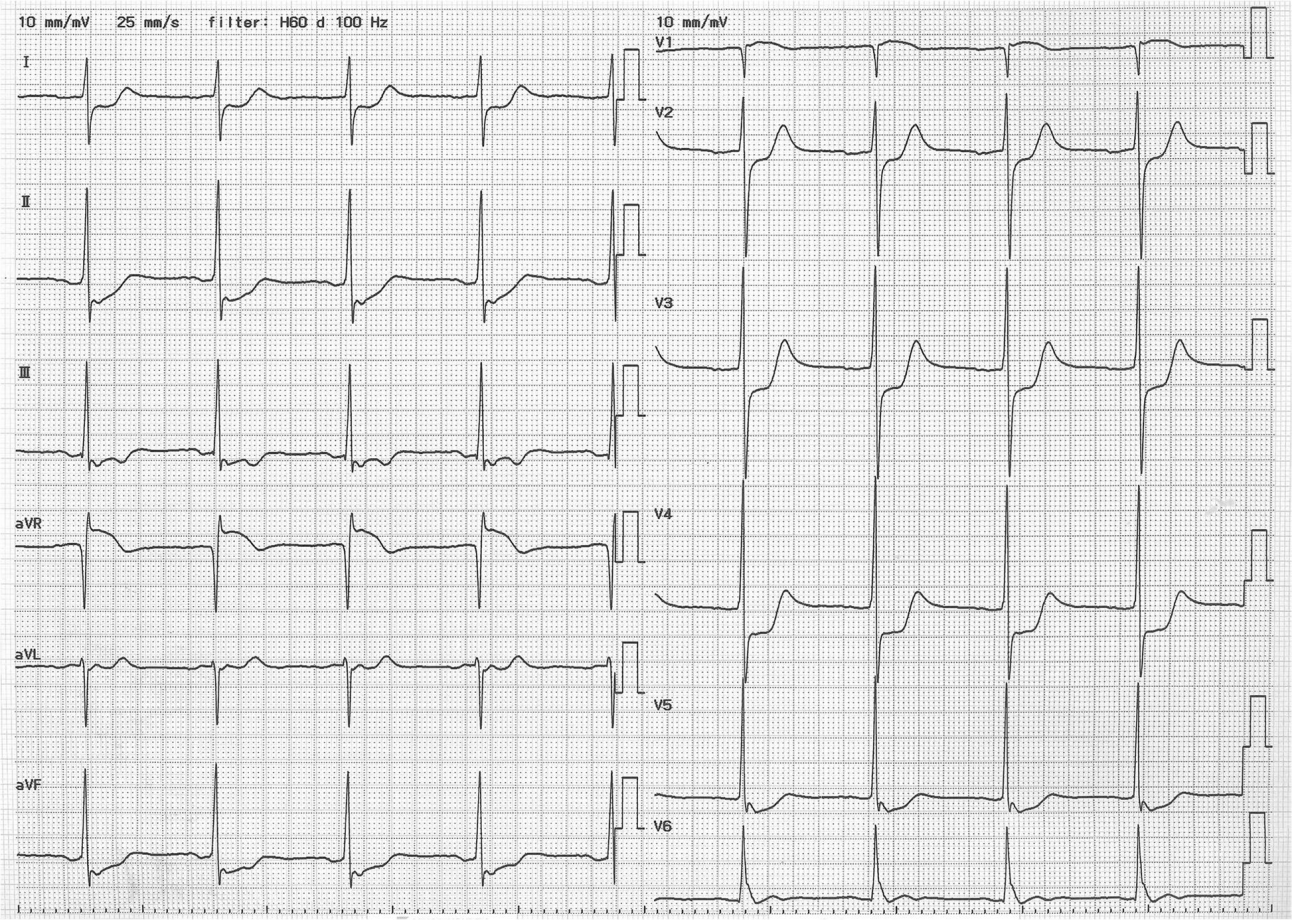
Electrocardiogram of case 4 shows ST segment depression in leads I–III, aVF, and V2–V5 and ST elevation in aVR

A 72-year-old man experienced a sudden severe headache with vomiting and called an ambulance. He was a heavy smoker and had a history of hypertension and Y-graft placement for an abdominal aortic aneurysm. When the emergency team arrived 10 min later, he was disoriented and incontinent of feces and urine. He did not complain of any chest symptoms. No obvious ST segment changes were noted on ECG during transportation to the hospital. On arrival, blood pressure was 106/82 mm Hg and heart rate was 64 bpm. He vomited and was intubated to secure the airway. ECG showed ST segment depression in leads I–III, aVF, and V2–V5 and ST elevation in aVR (Fig. 4). Echocardiography showed mitral regurgitation, akinesis of the posterior wall of the left ventricle, and hypokinesis in the anterior septum.

**Fig. 5:**
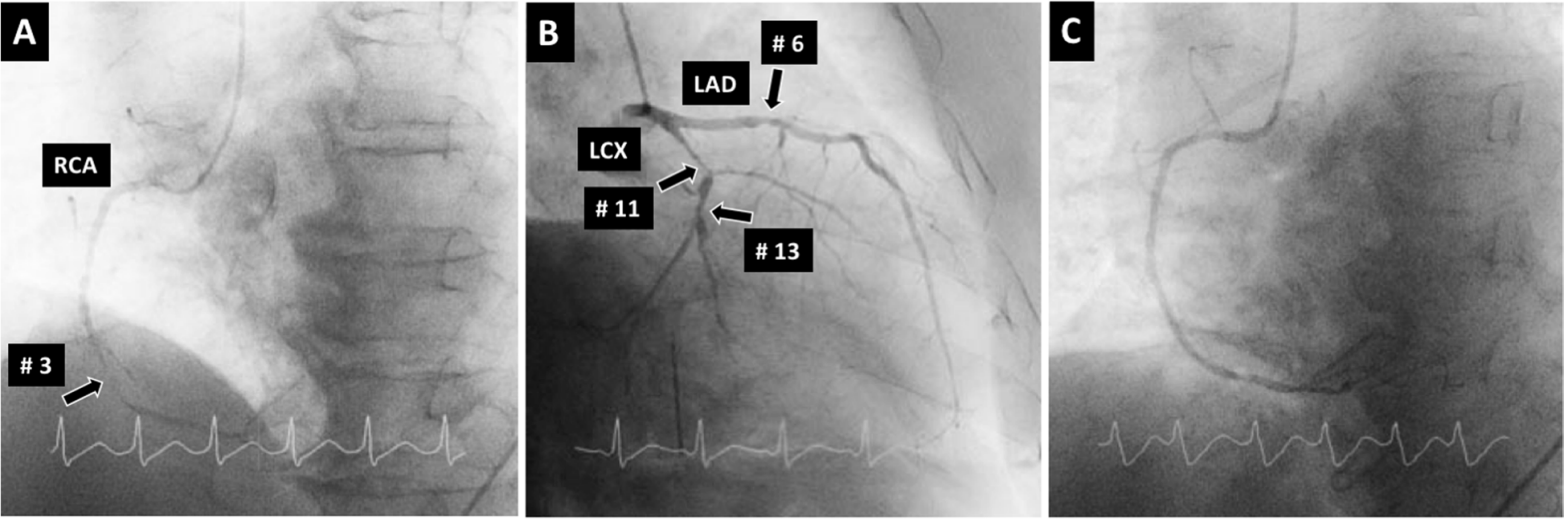
**A** Right coronary angiography of case 4 shows 99% stenosis of the right coronary artery segment 3. **B** Left coronary angiography shows 75% stenosis of the LAD segment 6 and 90% stenosis of the LCX segments 11 and 13. **C** Right coronary angiography after percutaneous coronary intervention with stenting to segment 3

SAH associated with neurogenic stunned myocardium was suspected and head CT was immediately performed; no significant lesions were found. CT angiography showed no cerebrovascular abnormalities. CKMB concentration was normal (1.6 ng/mL) but troponin T was positive. His blood pressure declined to 50 mm Hg/unmeasurable after CT and heart rate declined to 30 bpm. After vasopressor support was initiated, emergency CAG was performed and revealed 99% stenosis of the RCA segment 3, 75% stenosis of the LAD segment 6, and 90% stenosis of the LCX segments 11 and 13. The patient underwent PCI for segment 3, which was considered the culprit lesion (Fig. 5). Then, an intra-aortic balloon pump (IABP) was placed and he was transferred to the cardiovascular department as a potential candidate for mitral valve replacement. He underwent veno-arterial extracorporeal membranous oxygenation and the IABP was later replaced with a catheter-based miniaturized ventricular assist device. He did not complain of headache upon awakening but died 38 days later due to hemorrhagic complications.

### Literature review

The literature search initially identified 721 potentially relevant articles. Forty-eight articles including 55 cases of cardiac cephalalgia met criteria (Supplementary File 1). After including the four patients reported here, a total of 59 cardiac cephalalgia cases were finally reviewed. Individual patient characteristics are shown in Table 3 [13–54] and summarized in Table 4.

**Table 3:** Reported cases of cardiac cephalalgia

Author	Year	Age	Sex	Site	Quality	Intensity	Onset	Duration	Autonomic signs	Cardiac symptoms	Trigger	ECG	Coronary lesion	Therapy	Follow-up
Caskey [13]	1978	47	M	Right eye	Pressing	Severe	NA	30–40 s	None	Chest pain, l-arm pain	Rest, mild exercise	ST elevation	NA	Nitrate	Resolved
Lefkowitz [14]	1982	62	M	Bregmatic	Explosive	Severe	NA	NA	NA	Retrosternal pain, arm numbness	Stress, exertion	ST depression (stress)	3 vessel	CABG	Resolved
Fleetcroft [15]	1985	78	F	Frontal	NA	NA	NA	NA	None	Chest tightness	Mild exercise, cold, meal	ST elevation	NA	Nitrates	Resolved
Blacky [16]	1987	40	M	Bitemporal	NA	NA	NA	NA	None	None	Vigorous exercise	ST depression (stress)	RCA	Nitrates	Resolved
Vernay [17]	1989	71	M	Occipital parietal frontal	NA	NA	NA	NA	None	Shoulder pain radiating to arms	Exertion, exercise, meal	ST depression (stress)	NA	Nitrates	Resolved
Takayanagi [18]	1990	67	M	Occipital	pulsating	Severe	NA	a few minutes	None	Chest pressure	Hot bath, sleeping, urination	ST elevation	NA	Nitrates	Died
Takayanagi [18]	1990	64	F	NA	NA	NA	NA	NA	NA	Chest pain	NA	ST elevation	3 vessel	Nitrates	Died
Bowen [19]	1993	59	M	Bitemporal	NA	Severe	Sudden	10–30 m	None	Chest pressure, left arm pain	NA	ST depression	RCA, OM	PCI	Resolved
Ishida [20]	1996	64	M	Occipital	Throbbing	Severe	Sudden	10 h	Nausea	Shoulder pain	Rest	ST depression (stress)	3 vessel	PCI	Resolved
Lipton [2]	1997	57	M	Vertex	Sharp or shooting	Severe	Gradual	Minutes–hours	Nausea	Abdominal or chest pain	Vigorous exercise, sexual activity	ST depression (stress)	3 vessel	CABG	Resolved
Lipton [2]	1997	67	M	Bifrontal	Squeezy, steadily, pressing	Severe	Gradual	Minutes–hours	None	None	Vigorous exercise	ST depression (stress)	3 vessel	PCI	Resolved
Grace [21]	1997	59	M	Vertex occipital	Bursting	Severe	Sudden	Seconds	None	None	Mild exercise	ST depression (stress)	LAD, RCA	CABG	Relapse
Lance [22]	1998	62	M	Right frontal	NA	NA	Gradual	Minutes	None	Chest pain	Mild exercise	ST depression (stress)	LAD, RCA	CABG	Resolved
Lanza [23]	2000	68	M	Occipital	NA	NA	NA	NA	None	Shoulder pain	Rest	Peaked T in V2-4	3 vessel	CABG	Resolved
Lanza [23]	2000	70	M	Occipital	NA	NA	NA	NA	NA	None	Rest	NA	3 vessel	NA	NA
Amendo [24]	2001	78	F	Bitemporal	NA	Severe	NA	Hours	Vomiting	None	NA	ST elevation	3 vessel	CABG	Resolved
Amendo [24]	2001	77	F	Right frontal and maxillary	NA	Severe	Acute	Hours	None	None	NA	Precordial R progression	Normal	NA	NA
Auer [25]	2001	47	M	Occipital	NA	NA	NA	Minutes–2 h	NA	NA	NA	ST elevation	LAD, RCA	Advanced life support	Died
Rambihar [26]	2001	65	F	Occipital	NA	NA	NA	NA	NA	Shoulder and left arm pain	Exercise, meal	ST depression (stress)	3 vessel	CABG	Partially resolved
Famularo [27]	2002	70	M	Fronto-parietal bilateral	Sharp or shooting	Severe	NA	2 d	None	Mid epigastric pain	NA	ST elevation	NA	Nitrates	Resolved
Gutierrez-Morlote [28]	2002	59	M	Vertex occipital bilateral	Dull and throbbing	Moderate-severe	Rapidly progressive	1 d	Nausea, photophobia	Chest pain	Rest	ST depression	NA	Nitrates	Resolved
Martinez [29]	2002	68	F	Left hemicranial	Shooting	Severe	Gradual	1 h	None	None	Mild exercise, exertion	ST elevation	3 vessel	PCI	Resolved
Sathirapanya [30]	2004	58	M	Left occipital	Sharp or shooting	Severe	NA	15–20 m	None	Chest tightness	Exertion	ST elevation	3 vessel	CABG	Resolved
Chen [31]	2004	76	M	Bitemporal	Non-throbbing	Mild-severe	NA	5 m	None	Chest pain	Rest, exertion	ST depression (stress)	LAD, RCA	Nitrates	Resolved
Gutierrez-Morlote [32]	2005	74	F	Bitemporal	Pulsating	Severe	NA	Minutes–hours	Nausea	Chest tightness	Rest	ST depression	NA	Nitrates	Resolved
Gutierrez-Morlote [32]	2005	64	F	Uni- or bilateral	Oppressive	Severe	Sudden	1 h	None	None	Rest, mild exercise	NA	NA	NA	Died after resolution
Korantzopoulos [33]	2005	73	F	Occipital	Sharp	Severe	Sudden	1 h	Nausea, vomiting	None	Rest	ST depression	LAD	Nitrates	Resolved
Cutrer [34]	2006	55	M	Biparietal	Non-throbbing	NA	Gradual	Minutes	None	None	Mild exercise, sexual activity	Normal	LAD, RCA	PCI	Resolved
Seow [7]	2007	35	M	NA	Explosive	Severe	Gradual	1 d	Vomiting, cold sweating	None	NA	ST elevation	LAD	NA	Resolved
Broner [8]	2007	72	F	Occipital frontal bilateral	Sharp and throbbing	severe	Sudden	Hours	Nausea, vomiting pallor	None	Rest, exertion	ST elevation	RCA	Heparin	Resolved
Wei [35]	2008	36	M	Vertex to occipital bilateral	Dull	Severe	Rapidly progressive	NA	NA	NA	NA	ST elevation	LAD	PCI	Resolved
Wei [35]	2008	85	F	Right eye	NA	NA	NA	NA	NA	Chest pain	Exercise	NA	Normal	Nitrates	Resolved
Wang [36]	2008	81	F	NA	NA	Severe	NA	Hours	Dizziness, diaphoresis, nausea	VF	NA	ST elevation	RCX	PCI	Resolved
Dalzell [9]	2009	44	F	Occipital	NA	Severe	Sudden	NA	Nausea, vomiting, sweating	None	NA	ST elevation	RCA	PCI	Resolved
Sendovski [10]	2009	61	F	Forehead	NA	Severe	NA	NA	None	None	Exertion	ST depression	3 vessel	PCI	Resolved
Chatzizisis [37]	2010	42	M	Frontal bitemporal	NA	Severe	Sudden	Hours	None	None	NA	ST elevation	LAD	PCI	Resolved
Cheng [38]	2010	52	F	Bilateral	Throbbing	Severe	Sudden	3 d	None	Chest pain	Local anesthesia	Equivocal	Normal	Nitrates	Resolved
Cheng [38]	2010	67	F	Jaw, mandibula, bilateral temporoparietal	Throbbing	Severe	Sudden	5 m	None	Exertional dyspnea	Exertion	Normal	2 vessel	PCI	Resolved
Yang [39]	2010	44	F	Bifrontal	NA	Severe	NA	NA	Nausea	Chest tightness	Exertion	ST depression (stress)	spasm	Nitrates	Resolved
Costopoulos [40]	2011	55	M	Occipital	NA	NA	NA	NA	NA	None	Exertion	ST depression	3 vessel	Nitrates, CABG	Resolved
Elgharably [41]	2013	55	M	Frontal	NA	Severe	NA	> 12 h	None	None	NA	Q wave	LAD	PCI	Resolved
Asvestas [42]	2014	86	M	Occipital	NA	Severe	NA	NA	None	None	NA	ST depression	LCX, LAD	PCI	Resolved
Wassef [43]	2014	44	M	NA	Oppressive	Severe	NA	NA	None	Chest discomfort	Exertion	ST depression (stress)	LAD	PCI	Resolved
Mathew [44]	2015	47	M	Bioccipital to vertex	NA	Severe	NA	A few minutes	None	None	Exertion	NA	LAD	PCI	Resolved
Prakash [45]	2015	67	M	Posterior to holocephalic	Intense, excruciating	Severe	Sudden	10–60 m	Nausea	None	Lifting heavy objects, sexual activities	ST depression (stress)	3 vessel	CABG	Resolved
Chowdhury [46]	2015	51	M	Pre-auricula to forehead, vertex, occipital	NA	NA	NA	2–3 m	None	Mild chest tightness and sweating	Stress, exertion	Mild ST-T change	LAD, LCX	PCI	Resolved
Huang [47]	2016	70	F	Bilateral posterior nuchal	Dull squeezing	NA	Sudden	NA	Dizziness	None	None	ST elevation	LAD	PCI	Resolved
Shankar [48]	2016	73	M	Generalized	Dull	NA	NA	5 m	None	None	Exertion	ST depression (stress)	3 vessel	CABG	Resolved
Wang [6]	2017	40	M	Bitemporal	Pulsatile, tight	Moderate-severe	NA	5–10 m	Cold sweating	Chest discomfort, palpitations,	Exertion, cold stimuli, sexual activities	Inverted T	LAD, RCA, LCX, D	PCI	Resolved
Majumder [49]	2017	48	F	NA	NA	Severe	NA	Hours	None	None	Exertion	ST depression	LAD, RCA	PCI	Resolved
Lazari [50]	2019	64	M	Generalized	Compressing	Severe	Rapidly progressive	5–15 m	None	None	NA	ST elevation	RCA	PCI	Resolved
MacIsaac [51]	2019	86	M	Bilateral, posterior	Dull	Severe	Progressive	30–90 m	None	Chest pain	None	ST depression	RCA, LCX, D	Warfarin	Resolved
Santos [52]	2019	62	M	Holocranial	Aching	NA	NA	NA	None	Chest pain	None	Normal	3 vessel	CABG	Died
Ruiz Ortiz [53]	2020	74	F	Vertex, Bitemporal	Oppressive	Moderate	NA	NA	None	None	Exertion	ST elevation	3 vessel	PCI	Resolved
Sun [54]	2021	83	F	NA	Migraine-like	NA	NA	Hours	None	Chest pain	None	ST elevation	RCA	PCI	Resolved
Kobata	2021	69	M	Occipital	Pulsatile	Severe	Sudden	NA	Nausea, sweating	None	None	ST elevation	RCA	PCI	Resolved
Kobata	2021	66	F	Occipital	NA	Severe	Sudden	NA	Cold sweating	Chest discomfort	Exertion	ST elevation	LAD	PCI	Resolved
Kobata	2021	54	M	Occipital	Strangulation	Moderate	Gradual	NA	Cold sweating	Chest discomfort	Meal	ST elevation	3 vessel	PCI	Resolved
Kobata	2021	72	M	NA	NA	Severe	Sudden	NA	Nausea, vomiting	None	None	ST elevation	3 vessel	PCI	Resolved/Died

*M* male, *F* female, *NA* not available, *s* second, *m* minute, *h* hours, *d* day, *LAD* left anterior descending artery, *RCA* right coronary artery, *CX* circumflex artery, *OM* obtuse marginal artery, *D* diagonal artery, *CABG* coronary artery bypass graft, *PCI* percutaneous coronary intervention

**Table 4:** Clinical manifestations of cardiac cephalalgia

Characteristics		Variable (*N* = 59)
Age	Years (median, quartile)	64 (54–72)
Sex	Male	37 (62.7)
Triger	Exertion	26 (44.1)
	Other than exertion	3 (5.1)
	None	16 (27.1)
	NA	14 (23.7)
Onset	Sudden	15 (25.4)
	Progressive or gradual	11 (18.6)
	NA	33 (55.9)
Side	Right	4 (6.8)
	Left	2 (3.4)
	Bilateral	23 (39.0)
	NA	30 (50.8)
Regions	Frontal	10 (16.9)
	Temporal	7 (11.9)
	Parietal	3 (5.1)
	Occipital	23 (39.0)
	Whole	6 (10.2)
	Eye	2 (3.4)
	NA	8 (13.6)
Intensity	Severe	37 (62.7)
	Moderate-severe	2 (3.4)
	Moderate	2 (3.4)
	Mild -severe	1 (1.7)
	NA	17 (28.8)
Chest symptom	Present	24 (40.7)
	Absent	33 (55.9)
	NA	2 (3.4)
Associated symptoms	Nausea	10 (16.9)
	Sweating	7 (11.9)
	Vomiting	5 (8.5)
	Dizziness	2 (3.4)
	Miscellaneous	5 (8.5)
	None	1 (2.0)
	NA	8 (13.5)
ECG	ST elevation	23 (39.0)
	ST depression	9 (15.2)
	ST depression in stress	14 (23.7)
	Other changes	5 (8.5)
	Normal	4 (6.8)
	NA	4 (6.8)
Risk factors	Hypertension	21 (35.6)
	Diabetes	14 (23.7)
	Hyperlipidemia	19 (32.2)
	Smoking	20 (33.9)
	Obesity	4 (6.8)

Characteristics are shown as number (%) except for age

*NA*, not available

The vertex in the original description was classified as parietal

Cardiac cephalalgia generally occurs in middle-aged or older individuals (median age, 64 years) with male predominance (62.7%). Forty-seven patients (79.7%) were age 50 or older. Pain is typically triggered by varying degrees of exertion, sexual activity, and motion fluctuation (49.2%) but may develop at rest without any particular trigger (27.1%). Headache may occur suddenly or gradually increase in intensity. The most common location of the pain is the occipital region (39.0%), but it can occur in a variety of sites, most often bilaterally (39.0%).

The nature of the headache varies, which has been described as pulsating, throbbing, oppressive, bursting, or explosive. Regardless, the intensity is usually severe. Headaches are frequently associated with autonomic signs such as nausea, vomiting, and sweating. More than half of patients (55.9%) do not complain of chest symptoms, which makes diagnosis challenging. The reported duration of headache ranges from 30 s to a few days and they may occur intermittently for several years. Exertional headaches are almost always relieved by rest. SAH is suspected in cases of sudden severe headache and several patients underwent diagnostic lumbar puncture [2, 7–9, 24, 33, 39, 44].

ECG revealed ST segment elevation (39.0%), ST segment depression at rest (15.2%) or during stress testing (23.7%), and other abnormal findings (8.5%). ECG was normal or equivocal in four (6.8%). Among the 25 patients who underwent cardiac enzyme testing, the concentration was elevated in 21 patents (84%) and normal in four (16%).

Coronary risk factors were common: hypertension, smoking, hyperlipidemia, diabetes, and obesity were reported in 35.6%, 33.9%, 32.2%, 23.7%, and 6.8% of patients, respectively. Three patients, including one reported above, had a history of myocardial infarction or coronary intervention [28, 31]. These histories provide invaluable diagnostic clues.

Underlying cardiac pathology was AMI (50.8%), angina (47.5%), cardiomyopathy (1.7%), and not described (1.7%). CAG results were described in 51 patients. Coronary occlusion or severe stenosis was present in almost all patients. The number of affected arteries was three in 19 patients, two in 11, and one in 17; spasm was reported in two and findings were normal in two others.

PCI was performed in 26 patients and coronary artery bypass graft (CABG) in 12. Nitrates were administered in 15 patients, heparin in one, and warfarin in one. Advanced life support was performed in one patient because of cardiac arrest. Headaches resolved with improvement in myocardial ischemia. Nitroderivatives are effective and PCI or CABG leads to permanent resolution of the headache. Headache recurrence has been reported with restenosis of coronary arteries [21, 43, 46]. Overall, reported outcomes were as follows: headache resolution, 51; death, 6.; not reported, 2. Three patients died of cardiac failure or its complications, including one patient reported above [18, 52]. Two others died of VF [18, 25]. One died suddenly 6 months after headache onset [28]

## Discussion

This report presents four cases of cardiac cephalalgia that resulted from AMI. Two patients (cases 2 and 3) reported chest discomfort with associated triggers. In contrast, the other two (cases 1 and 4) presented with sudden severe headache that met the diagnostic criteria for a thunderclap headache without an identifiable trigger. The latter two lost consciousness after the headache and had no cardiac symptoms; therefore, SAH was initially suspected. After head CT confirmed no intracranial hemorrhage, emergency CAG was performed, followed by PCI. Notably, three patients presented with low blood pressure and one developed VF. Because all four exhibited abnormal findings on ECG and echocardiography, the diagnosis of cardiac cephalalgia was straightforward. Early cooperation with cardiologists enabled prompt cardiovascular examination and treatment. The headache resolved after successful coronary reperfusion in all cases.

In a study of 1546 AMI patients, headache was present (along with other symptoms) in 5.2% and was the primary complaint in 3.4% [55]. Differentiation of cardiac cephalalgia from migraine without aura has been emphasized in patients without chest symptoms. Vasoconstrictor medications (e.g., triptans, ergots) are contraindicated in patients with ischemic heart disease, while migraine-like headache may be triggered by angina treatments such as nitroglycerine [3].

The typical manifestation of cardiac cephalalgia is migraine-like pain on exertion. However, it may present as a thunderclap headache without a trigger, although this is not common. In a systemic review of thunderclap headache, more than 100 different causes were reported; cardiac cephalalgia was highlighted as an important causative systemic condition [56]. Above all, SAH is the most common cause of secondary thunderclap headache and should be the focus of initial assessment given its significant morbidity and mortality [57].

Early differentiation of SAH and AMI is crucial because both are potentially life-threatening. Rapid diagnosis and appropriate treatment are therefore critical. Because cardiac cephalalgia is not always associated with chest symptoms or ECG abnormalities, the diagnosis should be considered in middle-aged or older patients with coronary risk factors presenting with a first-episode headache.

Confusingly, SAH patients can also present with cardiac symptoms. ECG abnormalities are common in these patients and left ventricular wall motion abnormalities may develop in the absence of organic coronary artery stenosis. Echocardiography may show takotsubo-like or other types of abnormal wall motion. This manifestation is transient and has been called neurogenic stunned myocardium [58], which is often associated with hypotension and elevated myocardial enzyme concentration [59]. Accordingly, hypotension, ECG abnormalities, abnormal cardiac wall motion, and mildly elevated cardiac enzyme concentration do not preclude SAH. For patients with thunderclap headache, emergency head CT is indispensable; if no significant findings are detected, a cardiac workup should be initiated. ECG, echocardiography, measurement of cardiac enzyme concentrations, and coronary artery evaluation should be performed when cardiac cephalalgia is suspected.

Several mechanisms to explain the headache induced by myocardial ischemia have been hypothesized: 1) referred pain through the convergence of vagal afferents from the heart with trigeminal neurons in the spinal trigeminal nucleus or somatic afferents from C1–C3 in the upper spinothalamic tract [2, 4, 60]; 2) elevated intracranial pressure because of venous stasis resulting from ischemia-induced ventricular hypofunction and reduced cardiac output [2, 4]; 3) vasodilation within the brain secondary to myocardial ischemia-induced release of serotonin, bradykinin, histamine, and substance P [2, 4]; 4) presence of vasospasm in both coronary and cerebral arteries [4]; and 5) reversible contraction of microvessels or cortical spreading depolarization induced by cerebral hypoperfusion [6]. The last hypothesis is based on confirmation of cerebral hypoperfusion during a headache attack in the presence of normal cerebral arteries on MR angiography [6]. CT angiography and MR angiography in the patients reported here did not reveal constriction of visible cerebral arteries either.

Headache in cardiac cephalalgia does not present with uniform clinical characteristics. Some patients visit the outpatient clinic complaining of recurrent exertional headaches, while others are brought to the emergency room in shock or a comatose state. Cardiac symptoms may be absent and ECG may be normal, even with standard stress testing [34]. To diagnose cardiac cephalalgia, clinicians must be aware of it and also suspect its presence. Interestingly, among the 48 articles reporting cardiac cephalalgia, 28 were published in neurology journals and 17 in cardiovascular journals. This may reflect the fact that patients are usually initially seen by neurologists. Overlooked or delayed diagnosis can lead to serious consequences. First-line health care professionals should be aware of cardiac cephalalgia. When it is suspected, early collaboration with cardiologists is warranted.

## Conclusion

Cardiac cephalalgia resulting from AMI can present with or without chest discomfort and even mimic the classic thunderclap headache associated with SAH. It should be recognized as an emergency and treated without delay.

## Supplementary Information

**Additional file 1:** **Supplementary Figure 1.** PRISMA Flow diagram showing the database search algorithm.

## Data Availability

The datasets used and analyzed during the current study are available from the corresponding author on reasonable request.

## Abbreviations

ICHD-III: International Classification of Headache, Third Edition
SAH: Subarachnoid hemorrhage
PCI: Percutaneous coronary intervention
LAD: Left anterior descending artery
RCA: Right coronary artery
ECG: Electrocardiography
CT: Computed tomography
CKMB: Creatine kinase–myocardial band
CAG: Coronary angiography
AMI: Acute myocardial infarction
LCX: Left circumflex artery
VF: Ventricular fibrillation
MR: Magnetic resonance
IABP: Intra-aortic balloon pump
CABG: Coronary artery bypass graft
