# Supplementary material for: Cardiac cephalalgia: a case series of four patients and updated literature review
Source: Int J Emerg Med. 2022 Jul 29;15:33. doi: 10.1186/s12245-022-00436-2 (PMC9336087; doi:10.1186/s12245-022-00436-2)
Supplement: Supplementary file 1 — Additional file 1: Supplementary Figure 1. PRISMA Flow diagram showing the database search algorithm. [file 12245_2022_436_MOESM1_ESM.docx]

**Identification of studies via other methods**

**Identification of studies via databases and registers**

Records identified from:

Citation searching (n = 16)

Records removed *before screening*:

Duplicate records removed

(n = 25)

Records identified from:

Databases (n = 721)

**Identification**

Records screened

(n = 696)

Records excluded

(n = 653)

Reports not retrieved (n = 4)

Review (n = 2)

Editorial or comment (n=2)

Reports not retrieved

(n = 0)

Reports sought for retrieval

(n = 16)

Reports sought for retrieval

(n = 43)

**Screening**

Reports excluded:

(n = 0)

Reports excluded (n=7)

No case presentation (n = 6)

Not English (n = 1)

Reports assessed for eligibility

(n = 16)

Reports assessed for eligibility

(n = 39)

Reports of included studies

(n = 48)

**Included**

*From:*  Page MJ, McKenzie JE, Bossuyt PM, Boutron I, Hoffmann TC, Mulrow CD, et al. The PRISMA 2020 statement: an updated guideline for reporting systematic reviews. BMJ 2021;372:n71. doi: 10.1136/bmj.n71. For more information, visit: <http://www.prisma-statement.org/>
